# Heterologous Replicase from Cucumoviruses can Replicate Viral RNAs, but is Defective in Transcribing Subgenomic RNA4A or Facilitating Viral Movement

**DOI:** 10.3390/v10110590

**Published:** 2018-10-28

**Authors:** Shuangyu Gao, Jinda Lu, Xiaodong Cheng, Zhouhang Gu, Qiansheng Liao, Zhiyou Du

**Affiliations:** College of Life Sciences, Zhejiang Sci-Tech University, Hangzhou 310018, China; ssaiwnyg@163.com (S.G.); lujinda51@163.com (J.L.); hbndcxd@163.com (X.C.); hzgu@zstu.edu.cn (Z.G.); qshliao@aliyun.com (Q.L.)

**Keywords:** *Cucumovirus*, heterologous replicase, compatibility, transcription, subgenomic RNA, long-distance movement

## Abstract

Interspecific exchange of RNA1 or RNA2 between the cucumoviruses *cucumber mosaic virus* (CMV) and *tomato aspermy virus* (TAV) was reported to be non-viable in plants previously. Here we investigated viability of the reassortants between CMV and TAV in *Nicotiana benthamiana* plants by *Agrobacterium*-mediated viral inoculation. The reassortants were composed of CMV RNA1 and TAV RNA2 plus RNA3 replicated in the inoculated leaves, while they were defective in viral systemic movement at the early stage of infection. Interestingly, the reassortant containing TAV RNA1 and CMV RNA2 and RNA3 infected plants systemically, but produced RNA4A (the RNA2 subgenome) at an undetectable level. The defect in production of RNA4A was due to the 1a protein encoded by TAV RNA1, and partially restored by replacing the C-terminus (helicase domain) in TAV 1a with that of CMV 1a. Collectively, exchange of the replicase components between CMV and TAV was acceptable for viral replication, but was defective in either directing transcription of subgenomic RNA4A or facilitating viral long-distance movement. Our finding may shed some light on evolution of subgenomic RNA4A in the family *Bromoviridae*.

## 1. Introduction

Natural interspecific reassortant viruses containing heterologous replicase components have not been reported in viruses so far. One explanations could be that the heterologous replicase components are incompatible (e.g. with an absence of interaction) in directing the replication of viral RNAs in vivo. It is still unknown whether there are other factors limiting survival of such a reassortant.

*Cucumber mosaic virus* (CMV) and *tomato aspermy virus* (TAV) are taxonomically close species belonging to the genus *Cucumovirus* in the family *Bromoviridae* [1]. All members of *Bromoviridae* have a genome that is composed of three positive-sense, single-stranded RNA molecules, designated RNA1–3. Genomic RNAs can be translated directly as mRNA due to the presence of a m7-GpppG cap structure at their 5’ end and a tRNA-like structured at their 3’ end [2,3]. RNA1 encodes the 1a protein that contains a methyltransferase domain at its N-terminus and a helicase domain at its C-terminus; while RNA2 encodes the 2a protein (RNA-dependent RNA polymerase, RdRP) [1,4]. Proteins 1a and 2a are viral replicase components that form viral replication complex with host factors [5]. Molecular interaction between 1a and 2a is required for efficient viral replication [6,7,8]. Among the six genera of *Bromoviridae*, only the members of *Cucumovirus* and *Ilarvirus* express the 2b protein that is translated from RNA4A, the subgenomic RNA of RNA2 [9,10,11]. The CMV 2b protein is one of the best-studied RNA silencing suppressors [12,13,14]. It suppresses RNA silencing, mainly depending on its cytoplasm-localized portion by sequestering small RNAs to prevent the entry of the latter into the RNA-induced gene silencing complex (RISC) [14,15,16,17]. Viral symptoms induced by severe CMV strains are presumably attributed to the interference with host microRNA functions by CMV 2b proteins [18,19,20,21]. RNA3 is a bipartite mRNA encoding movement protein (MP) and coat protein (CP). The MP translated from RNA3 is responsible for viral cell-to-cell movement. The CP translated from RNA4 (the subgenomic RNA of RNA3) is a unique viral structure protein for packaging viral RNAs, also participating in viral long-distance movement and development of viral symptoms [1,4].

Recombination and reassortment of viral genomes are evolutionary events for viruses to obtain foreign genetic materials. Reassortment only occurs between the same or related viruses possessing segmented genomes. Reassortment within CMV strains has been studied extensively [4], indicating that all three genomic RNAs are interchangeable. However, several cases of interspecific reassortment demonstrate that RNA3 but neither RNA1 nor RNA2 are interchangeable within bromoviruses or cucumoviruses [22,23,24]. One such case is the reassortment between the *Bromovirus* species *brome mosaic virus* (BMV) and *cowpea chlorotic mottle virus* (CCMV) [22]. The heterologous combination of RNA1 and RNA2 from BMV and CCMV failed to replicate viral RNAs in barley protoplasts, presumably due to incompatibility of the heterologous replicase components [25]. Another case is the interspecific reassortment between two *cucumovirus* species, CMV and *peanut stunt virus* (PSV) [24]. The combination of PSV RNA1 and CMV RNA2 resulted in replicated genomic RNAs, but failed to transcribe subgenomic RNA4. Using yeast-2-hybrid assay, they detected the interaction between the C-terminal half of PSV 1a and the N-terminal half of CMV 2a, suggesting that the interaction between the heterologous replicase components was required for replication of genomic RNAs, but was not sufficient for transcription of subgenomic RNA4 [24].

Heterologous combination of RNA1 and RNA2 from CMV and TAV has been reported to be unsuccessful in replicating viral RNAs [23,26]. Interestingly, Masuta et al. [27] identified a hybrid reassortant that was composed of TAV RNA1, CMV RNA2 and RNA3, and a chimeric RNA containing CMV RNA2 and the 3’ 320 nucleotides of TAV RNA2. The 1a protein encoded by the reassortant had two amino acid mutations, which allowed it to interact with CMV 2a protein. In spite of the considerable information about the interchange between CMV and TAV, we here tested viability of all interspecific reassortants between CMV and TAV in *Nicotiana benthamiana* plants, and activity of heterologous replicase in replication and transcription of viral RNA. We found that the heterologous combination of the replicase components from both viruses was biologically active in directing viral RNA replication, but was defective in either transcribing subgenomic RNA4A or promoting viral long-distance movement. Our findings may shed some light on evolution of subgenomic RNA4A in the family *Bromoviridae*.

## 2. Materials and Methods

### 2.1. Plants and Viruses

*N. benthamiana* plants were grown under a 16-h photoperiod with a light intensity of 150 to 200 μE∙m^−2^∙s^−1^ at 23–25 °C. Bj-TAV was isolated from chrysanthemum plants grown in Beijing, China [28], and its genome has been sequenced previously [29].

### 2.2. Plasmid Construction

T-DNA-based infectious clones of Fny-CMV and Bj-TAV were generated by inserting full-length cDNAs of viral RNAs downstream of duplicated 35S promoter in the binary vector pCB301 according to the protocol described previously [30]. Briefly, the full-length cDNAs of Fny-CMV RNAs 1–3 were amplified using Q5 DNA polymerase (NEB) from the DNA constructs pFny109, pFny209, and pFny309 [31]. The amplified cDNAs were digested with *Bam*HI, and cloned into pCB301 pre-digested with *Stu*I and *Bam*HI, to generate pCB301-C1, pCB301-C2 and pCB301-C3, respectively. In the same way, we generated the infectious clones of Bj-TAV RNAs 1–3, designated pCB301-T1, pCB301-T2 and pCB301-T3, respectively. To express GFP in RNA3 of CMV or TAV, the CP sequence in pCB301-C3 or pCB301-T3 was replaced with enhanced GFP (eGFP) using the In-Phusion recombinant cloning kit (Takara, Tokyo, Japan) according to the manufacturer’s instruction, generating pCB301-C3-eGFP or pCB301-T3-eGFP, respectively. Using the same method, we replaced the 2b sequence in pCB301-C2 with eGFP, generating pCB301-C2-eGFP. The infectivity of the constructed clones was tested by inoculation of *N. benthamiana* plants via agroinfiltration.

To transiently express the replicase of CMV or TAV from a non-replicating transcript, cDNAs of 1a and 2a open reading frames (ORF) were amplified and cloned separately into pCB301 after digestion with *Stu*I and *Sac*I, generating pCB301-C1a, pCB301-C2a, pCB301-T1a, and pCB301-T2a. The 1a protein encoded by the members of *Bromoviridae* contains a methyltransferase domain (Met) at the N-terminal half and a helicase domain (Hel) at C-terminal half. To interchange the functional domain between C1a and T1a, we amplified four DNA fragments C1a-Met, C1a-Hel, T1a-Met, and T1a-Hel. Two full-length, chimeric 1a cDNAs, namely C-T and T-C, were generated by overlapping PCR using the mixture of C1a-Met and T1a-Hel, or T1a-Met and C2a-Hel as PCR templates, respectively. The resultant cDNAs were digested with *Stu*I and *Sac*I, followed by cloning into pCB301 to generate pCB301-C-T and pCB301-T-C. Prior to transformation into *Agrobacterium tumefaciens* GV3101, all constructs were authenticated by DNA sequencing.

### 2.3. Agrobacterium-Mediated Plant Inoculation

All T-DNA infectious clones were separately transformed into *A. tumefaciens* GV3101 using the CaCl_2_-mediated freeze–thaw method described previously [32]. Agrobacterium cells were grown in Luria–Bertani liquid medium containing 50 μg/mL kanamycin and 20 μg/mL rifampin at 28 °C in a shaker. *Agrobacterium*-mediated plant inoculation was performed according to the protocol described previously [30]. Briefly, cells carrying infectious clones of RNA1, RNA2, or RNA3 were mixed equally to make inoculum of CMV (C1C2C3), TAV (T1T2T3), or all six reassortants designated C1C2T3, T1T2C3, and so on. The cell mixtures were infiltrated into the fourth and fifth true leaves of *N. benthamiana* plants. Mock plants were infiltrated with infiltration solution (10 mM MgCl_2_, 10 mM 2-(N-morpholino) ethanesulphonic acid and 100 mM acetosyringone). The plants were photographed at 7 days post-infiltration (dpi). Viral infection was analyzed through detection of progeny viral RNAs in the upper systemic and/or inoculated leaves by RNA gel blot.

### 2.4. Viral Replication Assays

To test the effect of the 1a proteins of CMV and TAV on replication of CMV RNA2 and RNA3, *Agrobacterium* cells carrying either pCB301-C1a or pCB301-T1a were equally mixed with the cells carrying pCB301-C2, pCB301-C3, and 35S-p19 (p19, a RNA silencing suppressor) [33], and infiltrated into the fifth and sixth true leaves of *N. benthamiana* plants. Meanwhile, the leaves infiltrated with infiltration solution or the cells carrying pCB301-C2 and pCB301-C3 were used as negative controls. At 5 dpi, progeny viral RNAs were analyzed by RNA gel blot. CMV 2b and CP proteins were analyzed by immunoblot.

To test compatibility of the heterologous replicase, *Agrobacterium* cells carrying a pair of pCB301-C1a and pCB301-T2a, or pCB301-T1a and pCB301-C2a were mixed with the cells carrying pCB301-C3-eGFP or pCB301-T3-eGFP equally. 35S-p19 was included in this assay. The mixed cells were infiltrated into the fifth and sixth true leaves of *N. benthamiana* plants. Mock plants were inoculated with infiltration solution. GFP fluorescence was recorded under UV light using a Nikon digital camera at 5 dpi.

To determine the role of the 1a protein in directing transcription of subgenomic RNA4A, *Agrobacterium* cells harboring pCB301-C2-eGFP were mixed equally with the cells containing pCB301-C2a and the construct expressing the wildtype or chimeric 1a protein. The p19 protein was co-expressed to suppress RNA silencing in this assay. Mock plants were infiltrated with infiltrating solution. Agroinfiltration and visualization of GFP fluorescence was performed as described above. At 5 dpi, GFP expression was analyzed by immunoblot, and C2-eGFP and its subgenomic RNA were detected by an RNA gel blot.

### 2.5. RNA Gel Blot Analysis

Total RNA was extracted from 0.1-g leaf tissues using RNA extracting solution (0.05 M sodium acetate, pH 5.2, 10 mM EDTA pH 8.0, 1% SDS). An RNA gel blot was carried out using the procedure described previously [15]. A digoxigenin (DIG)-labeled DNA oligonucleotide (probe-I40) complementary to a conserved sequence at the 3’ end region of all genomic RNAs in cucumoviruses [34] was used to probe all RNAs of CMV and TAV simultaneously. To specifically detect viral RNAs of CMV or TAV, we used two DIG-labeled DNA oligonucleotides: a CMV-specific probe (5’GGACCGAAGTCCTTCCGAAGAAACCTAGGAGATGGTTTCAA3’) and a TAV-specific probe (5’ CCGGGATAAAGGGTTCAAAGGCACCTCATCGATCAAAGAC3’). To specifically detect CMV subgenomic RNA4A, we used a DIG-labeled DNA oligonucleotide (CMV2b-specific probe) that is completely complementary to the sequence at positions 2589–2627 of CMV RNA2. The DIG-labeled probes were detected using a DIG High Prime DNA Labeling and Detection Starter Kit II (Roche, Basel, Switzerland) according to the manufacturer’s instruction.

### 2.6. Immunoblotting Analysis

Detection of GFP, CMV 2b or CP was performed as described previously [35]. Briefly, total protein was extracted from 0.2 g leaf tissues using phosphate-buffered saline (0.14 M NaCl, 0.01 M potassium phosphate, pH 7.4) supplemented with 2% (*v*/*v*) 2-mercaptoethanol, and separated in SDS-polyacrylamide gel electrophoresis, followed by transfer to nitrocellulose membranes (GE, Chicago, IL, USA). The membranes were probed using polyclonal anti-GFP (Santa Cruz, CA, USA), anti-2b [16], or anti-CP serum [36], and primary antibody binding was detected using horseradish peroxidase-conjugated anti-rabbit or anti-mouse IgG (Santa Cruz) and a chemiluminescence reagent kit (Thermo-Fisher, Waltham, MA, USA) according to the procedure described previously [35].

## 3. Results

### 3.1. Effect of Genomic Reassortment on Virus Infection

**Figure 1:**
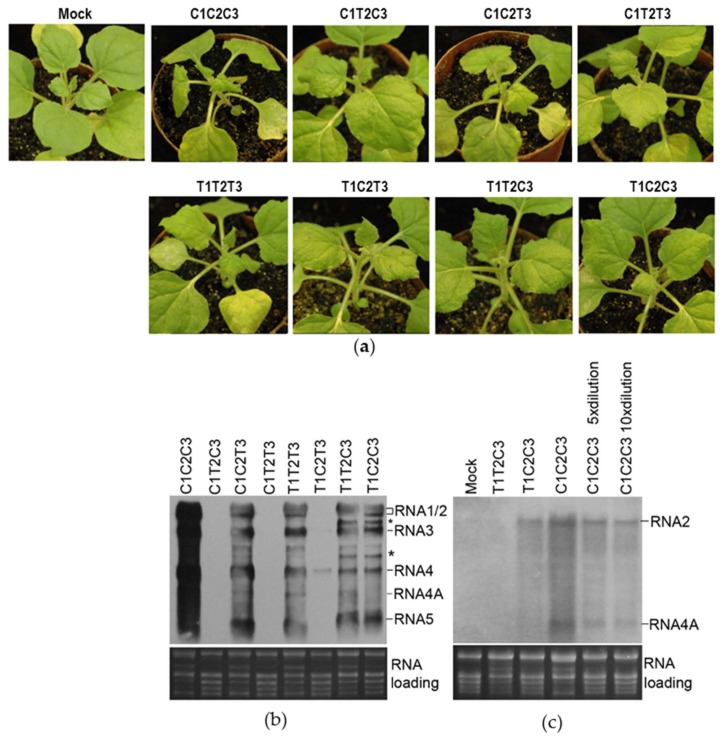
Infection by *cucumber mosaic virus* (CMV), *tomato aspermy virus* (TAV) and their reassortants in plants. (**a**) Viral symptoms on *Nicotiana benthamiana* plants with virus inoculation. Photographs were taken at 7 days post-infiltration (dpi). C1–3 and T1–3 represent genomic RNA1–3 of CMV and TAV, respectively. (**b**) Northern blot analysis of progeny viral RNAs in the upper systemic leaves harvested at 7 dpi. The probe was a DIG-labeled oligonucleotide that hybridizes all RNAs of CMV and TAV. Asterisk (*) shown on the right indicates the extra bands presented in T1T2C3 and T1C2C3. Ribosomal RNAs were used as a loading control. (**c**) Northern blot analysis of RNA2 and its subgenomic RNA4A in the RNA samples indicated. Here, 5× or 10× dilution indicates 5-fold or 10-fold dilutions of total RNA of C1C2C3 with the mock RNA sample, respectively. The probe used here was a DIG-labeled oligonucleotide specific for the CMV 2b sequence. Ribosomal RNAs were used as a loading control.

To test the effect of genomic reassortment on virus infection, we inoculated all viral reassortants between CMV and TAV, as well as the wildtype viruses CMV (C1C2C3) and TAV (T1T2T3) in *N. benthamiana* plants through agroinfiltration. At 7 dpi, CMV-inoculated plants showed obvious viral symptoms, such as severe distortion of the top leaves, and the plants inoculated with TAV or C1C2T3 displayed moderate symptoms. However, all other inoculated plants showed mild or no symptoms (Figure 1a). To determine viral infection, progeny viral RNAs were tested in the upper systemic leaves by RNA gel blot using a probe that can hybridize all RNAs of CMV and TAV. CMV-infected plants showing the most severe symptoms had dramatically high level of viral RNAs. The reassortants with exchange of RNA3 (C1C2T3 and T1T2C3) were detected, verifying that the exchange of RNA3 is allowable between the two viruses. However, the reassortants C1T2C3 and C1T2T3 were undetectable, suggesting that the C1T2 combination was defective in either viral replication, systemic movement, or both. In the case of combination between TAV RNA1 and CMV RNA2 (T1C2), the reassortant T1C2C3 accumulated at a level, as did TAV, whereas another reassortant T1C2T3 showed limited RNA3 and its subgenomic RNA4. The difference in virus accumulation between T1C2C3 and T1C2T3 demonstrates that RNA3s of CMV and TAV had a differential response to the T1C2 combination.

Virulence caused by CMV or TAV mainly depends on RNA silencing suppressor 2b that is expressed from subgenomic RNA4A [37,38]. As shown above, T1C2C3 infected the plants systemically, but did not cause discernable symptoms (Figure 1a). We noticed that the reassortant produced RNA4A at an undetectable level (Figure 1b). RNA4A was still not detected in T1C2C3 using a CMV2b-specific probe (Figure 1c). However, RNA4A could be detected in the 10-fold diluted RNA sample of C1C2C3 that showed a level of RNA2 as did T1C2C3 (Figure 1c). All these results demonstrate that the heterologous combination of TAV RNA1 and CMV RNA2 was defective in synthesis of CMV subgenomic RNA4A, which may be an explanation for the absence of viral symptoms in the T1C2C3-infected plants.

### 3.2. Heterologous Combination of CMV RNA1 and TAV RNA2 was Defective in Viral Long-Distance Movement

**Figure 2:**
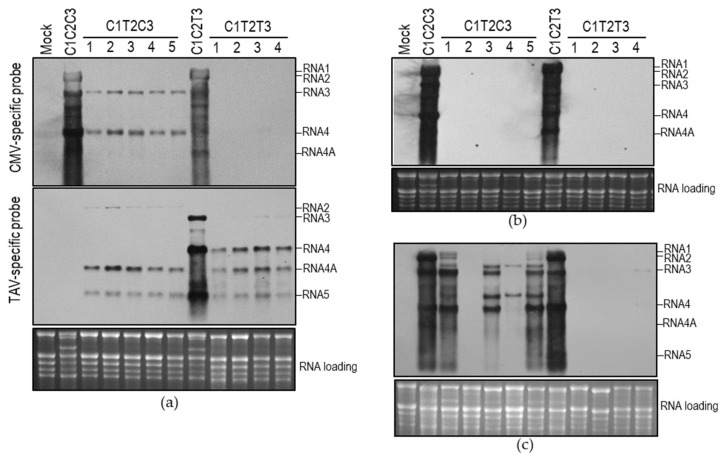
The reassortants containing C1T2 were defective in viral long-distance movement in plants. Numbers (1–5, or 1–4) shown on the figure indicate five or four individual plants inoculated with C1T2C3 or C1T2T3, respectively. Viral RNAs were analyzed in the inoculated leaves at 5 days post-infiltration (dpi) (**a**) and in the upper systemic leaves at 7 dpi (**b**) and 14 dpi (**c**) by Northern blot. The digoxigenin (DIG)-labeled probes used in panel (**a**) are shown on the left. The probe used in the panels (**b**,**c**) was a DIG-labeled oligonucleotide common for *cucumber mosaic virus* and *tomato aspermy virus* (TAV). Ribosomal RNAs were used as a loading control.

As shown above, C1T2C3 and C1T2T3 were undetectable in the upper systemic leaves at 7 dpi (Figure 1b), which can be attributed to defects in either viral replication or long-distance movement. To distinguish the possibilities, we analyzed accumulation of viral RNAs of both reassortants in the inoculated and upper systemic leaves of five or four individual, virus-inoculated plants, respectively, by RNA gel blot. At 5 dpi, all viral RNAs of the two reassortants were detected in the inoculated leaves using CMV-specific or TAV-specific probe separately, with exceptions of CMV RNA1 in the C1T2C3- or C1T2T3-inoculated plants, and TAV RNA2 in the C1T2T3-inoculated plants (Figure 2a). Failure to detect those viral RNAs may be due to low accumulation levels under detection limit. These results suggest that both reassortants were viable in the inoculated leaves, although they accumulated at a much lower level than C1C2C3 or C1C2T3. At 7 dpi, viral RNAs were not detected in the upper systemic leaves of all these plants inoculated with C1T2C3 or C1T2T3 using a common probe for both CMV and TAV (Figure 2b). At 14 dpi, C1T2T3 was nearly undetectable in these four plants, while C1T2C3 was detected, with varied accumulation levels in four plants (No. 1, 3, 4, 5) (Figure 2c). Failure of C1T2C3 to infect the plant (number 2) systemically suggests that C1T2C3 was defective in its systemic movement, although other four plants were infected systemically. Systemic infection of C1T2C3 in these four plants could be due to occurrence of genetic alteration to viral genome. Indeed, we observed two additional RNA bands commonly presenting in these four infected plants. Sequencing 3’ terminal part of RNA3 from No. 4 plant indicates that the extra RNA bands were chimeric RNA3 and its subgenomic RNA4 containing an exact 3’UTR of TAV RNA2 at their 3’ termini. The chimeric RNA3 may be a causative that allows C1T2C3 to move systemically in the plants. Taken together, our data suggest that the heterologous combination of CMV RNA1 and TAV RNA2 was defective in facilitating viral long-distance movement.

### 3.3. Heterologous Replicase Components were Compatible in Replication of GFP-Expressing RNA3

**Figure 3:**
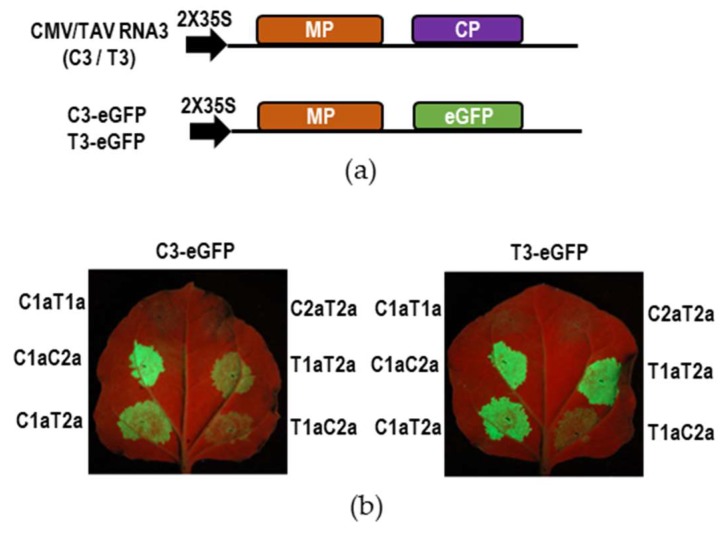
Replication of Green fluorescence protein (GFP)-expressing RNA3 promoted by the heterologous replicase from *cucumber mosaic virus* (CMV) and *tomato aspermy virus* (TAV). (**a**) Schematic diagram of the infectious clones for the wildtype or GFP-expressing RNA3 of CMV or TAV controlled by a double-35S constitutive promoter. C3-eGFP and T3-eGFP were generated by substitution of their coat protein (CP) sequence with enhanced GFP in the infectious clone of CMV or TAV RNA3, respectively. (**b**) Visualization of GFP fluorescence in the leaves of *Nicotiana benthamiana* plants expressing the homologous or heterologous replicase together with C3-eGFP or T3-eGFP as a replicating template. RNA silencing suppressor p19 was co-expressed to inhibit plant antiviral RNA silencing in the assay. Photographs were taken under UV light at 5 days post-infiltration.

To test compatibility of the heterologous replicase components between CMV and TAV, the homologous and heterologous replicases were expressed from the non-replicating constructs to promote replication and transcription of the GFP-expressing RNA3s, C3-eGFP and T3-eGFP (Figure 3a). GFP fluorescence density is considered as an indicator of the RNA4 level transcribed from C3-eGFP or T3-eGFP, and generally correlates with the replication level of the modified RNA3. The results obtained from the visualization of GFP fluorescence at 5 dpi showed that except for both negative controls C1aT1a (CMV 1a plus TAV 1a) and C2aT2a (CMV 2a plus TAV 2a), all other pairs of replicase produced GFP fluorescence to a varied extent. Both homologous pairs C1aC2a and T1aT2a produced similarly strong GFP fluorescence from their homologous RNA3 variant. C1aC2a was equally efficient as T1aT2a in replicating T3-eGFP, with the appearance of similarly strong GFP fluorescence, but the latter was less efficient than the former in producing GFP from C3-eGFP. The heterologous pair C1aT2a produced strong GFP fluorescence from either RNA3 variant as did both homologous pairs, suggesting that the heterologous combination of CMV 1a and TAV 2a was compatible in directing replication and transcription of viral RNAs, at least of RNA3. The reciprocal pair T1aC2a produced similar intensity of GFP fluorescence from C3-eGFP and T3-eGFP, which was obviously weaker than that produced by C1aT2a. This suggests that T1aC2a was compatible at least partially in replication and transcription of RNA3.

### 3.4. Heterologous Combination between TAV 1a and CMV 2a was Deficient in Synthesis of CMV Subgenomic RNAs

**Figure 4:**
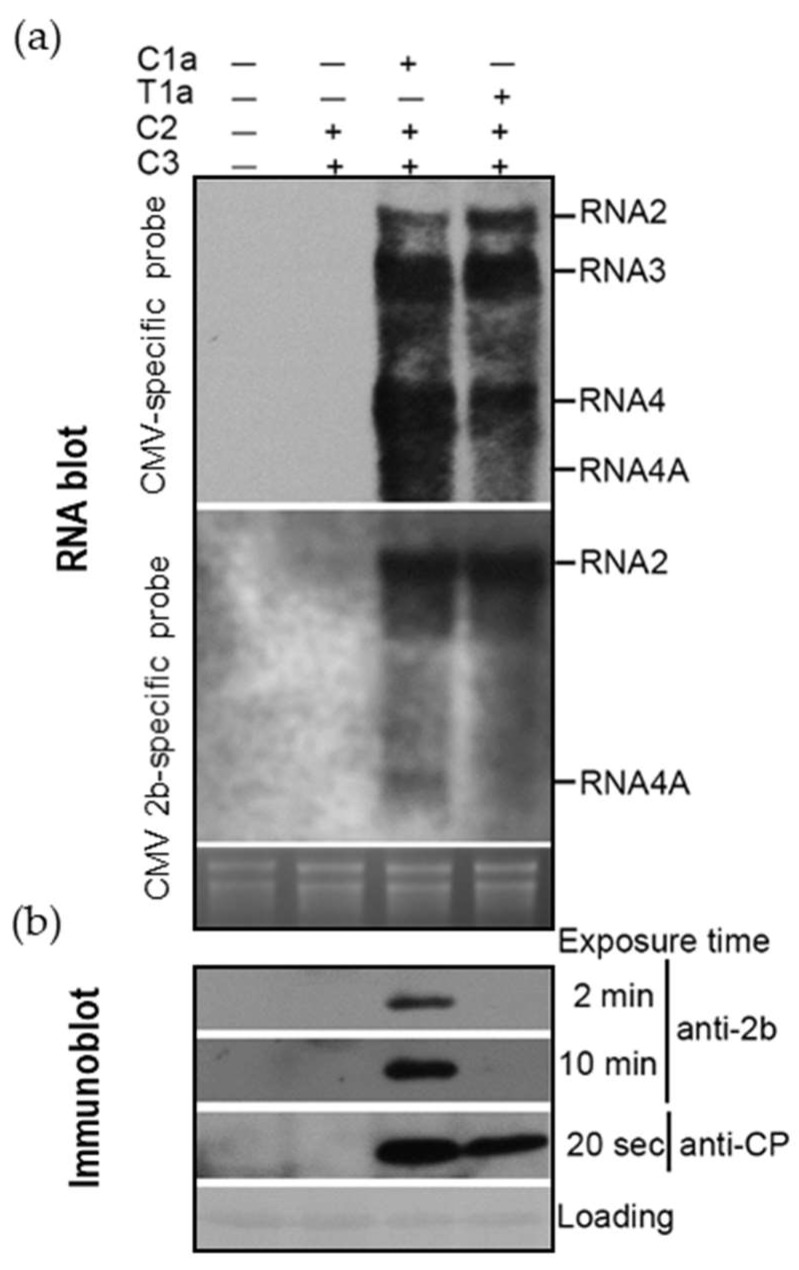
The combination of *tomato aspermy virus* (TAV) 1a and *cucumber mosaic virus* (CMV) 2a was deficient in transcribing subgenomic CMV RNAs. CMV RNA2 and RNA3 were transiently expressed together with 1a of TAV or CMV in the leaves of *Nicotiana benthamiana* plants through agroinfiltration. RNA silencing suppressor p19 was transiently co-expressed in this assay. (**a**) Northern blotting analysis of viral RNAs using either a CMV- or CMV 2b-specific probe, shown on the left. Total RNAs were extracted from the infiltrated leaves at 5 days post-infiltration. Ribosomal RNAs were used as a loading control. (**b**) Immunoblotting analyses of CMV 2b and CP in the infiltrated leaves. Ponceaus S staining was used to monitor equivalence of protein loading and transfer.

As shown in Figure 1b, T1C2C3 produced RNA4A from CMV RNA2 at an undetectable level in the plants. To determine whether the 1a protein encoded by TAV RNA1 was responsible for the defect, the 1a protein of CMV or TAV was transiently co-expressed with CMV RNA2 and RNA3 in the leaves of *N. benthamiana* plants through agroinfiltration. Northern blot analysis of viral RNAs showed that C2 and C3 could not replicate at a detectable level in the absence of either 1a protein, verifying that the 1a protein is essential for efficient viral replication (Figure 4a). When provided, both 1a proteins promoted replication of C2 or C3 at a similar level. However, TAV 1a showed lower efficiency in producing RNA4 than CMV 1a. As expected, CMV 1a but not TAV 1a directed transcription of RNA4A, which was detected by CMV-specific probe (upper panel) or CMV 2b-specific probe (lower panel). Immunoblotting data showed that the 2b protein expressed from CMV RNA4A was detected in the presence of CMV 1a, but not TAV 1a (Figure 4b). Moreover, CMV CP translated from RNA4 accumulated in CMV 1a-expressing leaves more than that in TAV 1a-expressing leaves (Figure 4b). All these data demonstrate that the heterologous combination between TAV 1a and CMV 2a was deficient in transcribing CMV subgenomic RNAs. In other words, TAV protein 1a was more selective in transcribing CMV sgRNAs than CMV 1a.

**Figure 5:**
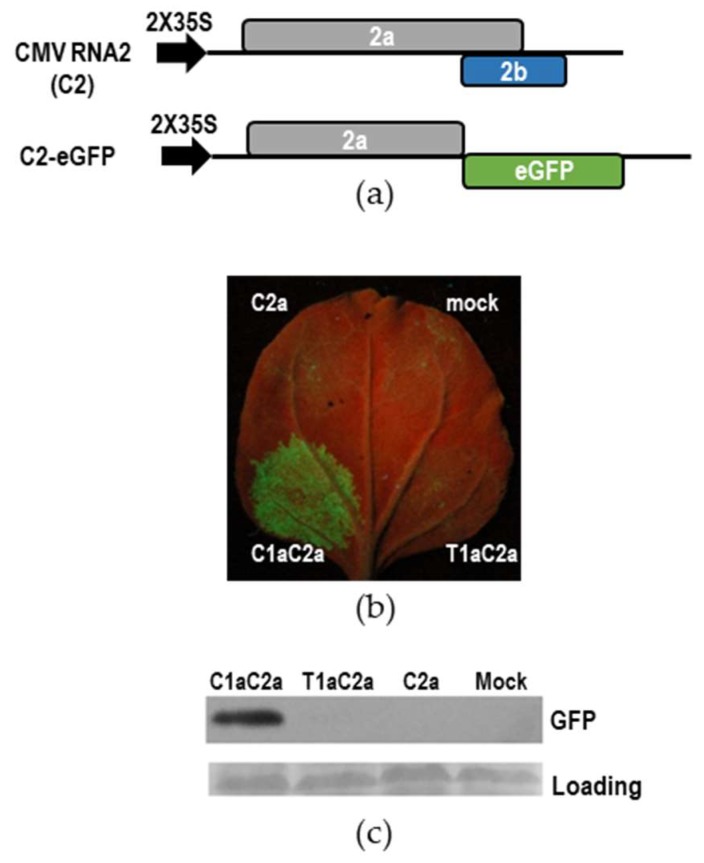
The combination of *tomato aspermy virus* (TAV) 1a and cucumber mosaic virus (CMV) 2a was defective in transcribing subgenomic RNA from green fluorescence protein (GFP)-expressing RNA2. (**a**) Schematic diagram of the infectious clones for the wild-type (C2) or GFP-expressing (C2-eGFP) RNA2 of CMV. C2-eGFP was generated by replacing the 2b open reading frame (ORF) with eGFP, and pre-maturely terminating the 2a ORF in the C2 construct. (**b**) Visualization of GFP fluorescence in the infiltrated leaves of *Nicotiana benthamiana* plants. C2-eGFP was co-expressed with the combination of C1a and C2a, T1a and C2a, or C2a alone. The mock patch expressed C2-eGFP alone. RNA silencing suppressor p19 was transiently co-expressed in this assay. GFP fluorescence was recorded under UV light at 5 days post-infiltration. (**c**) Immunoblotting analysis of GFP accumulation in the infiltrated patches. Ponceau S staining was used to monitor equivalence of protein loading and transfer.

To better demonstrate the essential role of CMV 1a protein in synthesis of CMV RNA4A, we tested the transcription of GFP-expressing CMV RNA2 (C2-eGFP) promoted by C2a with C1a or T1a through agroinfiltration. In C2-eGFP, the 2b ORF is replaced with eGFP and the 2a ORF is pre-maturely terminated (Figure 5a). Visualization of GFP fluorescence showed that strong fluorescence was observed in the patch co-expressing C1a and C2a, but no fluorescence was observed in the patches that expressed T1a and C2a, or C2a alone, or in the mock patch (Figure 5b). Immunoblotting analysis of GFP showed that GFP was detected only in the patch co-expressing C1a and C2a (Figure 5c). All evidence supports the conclusion that CMV 1a protein was essential for efficient synthesis of CMV RNA4A, and the heterologous combination of TAV 1a and CMV 2a was not compatible in this event.

### 3.5. The Helicase Domain of 1a was Required for Synthesis of CMV RNA4A

**Figure 6:**
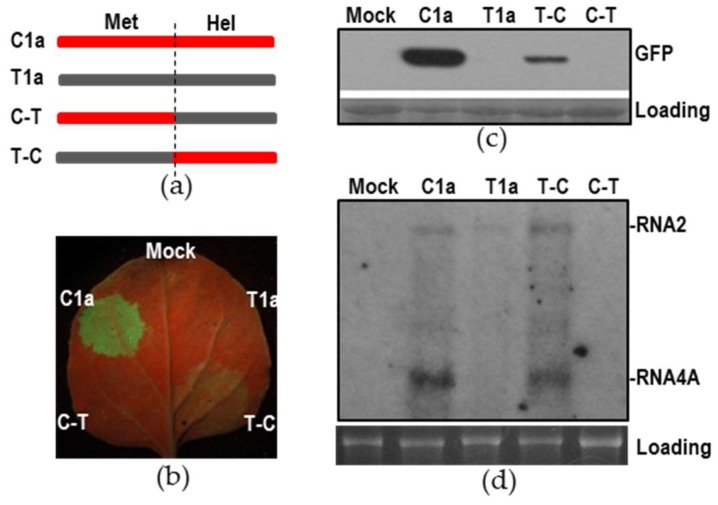
The C-terminal helicase domain of cucumber mosaic virus (CMV) 1a was required for synthesis of subgenomic RNA4A. (**a**) Schematic diagram of the interchange of the functional domains between the 1a proteins of CMV and *tomato aspermy virus*. Met and Hel indicate methyltransferase domain and helicase domain, respectively. (**b**) Visualization of green fluorescence protein (GFP) fluorescence in the infiltrated leaves of *Nicotiana benthamiana* plants. CMV 2a was transiently co-expressed with either wildtype or chimeric 1a protein to replicate C2-eGFP (See Figure 5a for schematic diagram) through agroinfiltration. RNA silencing suppressor p19 was co-expressed in this assay. GFP fluorescence was photographed under UV light at 5 days post-infiltration (dpi). (**c**) Immunoblotting analysis of GFP accumulation in the infiltrated leaves at 5 dpi. Ponceau S staining was used to monitor equivalence of protein loading and transfer. (**d**) Northern blotting analysis of C2-eGFP and its subgenomic RNA in the infiltrated leaves using a CMV-specific probe. 25S rRNA was used as a loading control.

Protein 1a encoded by the members of *Bromoviridae* contains a methyltransferase domain at its N-terminus and a helicase domain at its C-terminus. To determine which domain of the 1a protein was required for synthesis of CMV RNA4A, an interchange was made between C1a and T1a to generate two chimeric 1a proteins: C-T containing an N-terminus of CMV 1a and a C-terminus of TAV 1a, and T-C, the opposite to C-T (Figure 6a). Then, we tested replication of C2-eGFP initiated by CMV 2a with the wildtype or chimeric 1a proteins together. At 5 dpi, like T1a, C-T did not produce GFP fluorescence. In contrast, T-C produced visible GFP fluorescence, although the fluorescence was much weaker than that produced by CMV 1a (Figure 6b). The immunoblotting data of GFP is consistent with the observation of GFP fluorescence (Figure 6c). Northern blotting analysis showed that the subgenomic RNA transcribed from C2-eGFP was detected in the presence of C1a or the chimeric 1a T-C (Figure 6d), which is consistent with the results of fluorescence observation and immunoblot. Unexpectedly, C-T failed to produce detectable C2-eGFP and its sgRNA, suggesting that C-T was inactive in directing C2-eGFP replication. Collectively, the helicase domain of CMV 1a protein was required for synthesis of CMV RNA4A.

## 4. Discussion

Our work demonstrates that the exchange of RNA1 or RNA2 between CMV and TAV was acceptable for viral RNA replication in plants. However, the exchange was deficient in either promoting viral long-distance movement or directing transcription of sgRNAs, suggesting that the heterologous replicase was compatible in viral replication, but not competent to transcribe sgRNAs and facilitate viral movement.

It was reported previously that the exchange of RNA1 or RNA2 between CMV and TAV was unsuccessful in infection of *Chenopodium amaranticolor* by mechanically inoculating viral RNAs isolated from virions, demonstrating the incompatibility of the heterologous RNA1 and RNA2 from the two viruses [23]. Here, we found that the heterologous combination of RNA1 and RNA2 from both viruses could infect *N. benthamiana* plants locally or systemically (Figure 1 and Figure 2). The distinction observed between our work and that of Rao and Francki may be attributed to the different inoculation methods. Compared with mechanical inoculation of viral RNAs, *Agrobacterium*-mediated virus inoculation used in our work would have a higher infection efficiency as it allows inoculation of more adjacent cells and also continues synthesis of viral RNAs from the plasmids delivered by *Agrobacterium* cells. The advantage has made agroinfiltration the standard inoculation method in laboratories so far. However, the advantage may raise a possibility that agroinfiltration provides a window to allow occurrence of additional mutagenesis to viral RNA transcripts. Thus, we cannot rule out the possibility that agroinfiltration introduced additional mutations to allow better compatibility between the heterologous replicase of CMV and TAV in our assays.

Incompatibility of heterologous replicase was supposed to be absent of molecular interaction between the heterologous replicase components [24,25,27]. Masuta et al. [27] found that TAV 1a with mutations obtained the ability to interact with CMV 2a, which was supposed to be an explanation for the infection of a quadripartite hybrid virus in tobacco plants. In our work, the heterologous replicase from CMV and TAV would be compatible at least in replication as they replicated the wildtype or modified RNA2 or RNA3 efficiently in plants (Figure 3, Figure 4 and Figure 5), especially the combination of C1a and T2a, showing similar replication efficiency to the homologous replicase (Figure 3). In our replication assays, proteins 1a and 2a were expressed from non-replicating transcripts, which allows us to rule out the possibility that compatibility of the heterologous replicase from the two viruses was the consequence of additional mutations introduced by agroinfiltration. We believe different isolates or strains of viruses may be distinct in compatibility of heterologous replicase, and it would be necessary to test more isolates or strains to determine compatibility of interspecific replicase complex.

Allison et al. [22] reported that the exchange of RNA3 between BMV and CCMV was defective in viral systemic movement in their natural hosts. The same situation did not occur in the exchange of RNA3 between CMV and TAV or PSV (Figure 1b) [23,24]. We observed that although C1T2C3 and C1T2T3 replicated in the inoculated leaves (Figure 2a) and their replicase components (C1a and T2a) replicated GFP-expressing RNA3 efficiently (Figure 3), they were unable to infect the plants systemically at the early stage of infection (Figure 2b), indicating that the replicating compatibility between CMV RNA1 and TAV RNA2 was not sufficient to mediate viral movement. In addition to CP and MP, CMV 1a and 2a proteins have been reported to function in viral movement in plants [39,40,41]. Thus, the failure of C1T2C3 and C1T2T3 to infect plants systemically could be due to improper interaction among MP, CP and replicase, and/or viral genomes.

Some evidence demonstrates that the combination of TAV 1a and CMV 2a was inefficient in transcribing CMV RNA4 (Figure 3 and Figure 4). Similar events have been reported previously by Suzuki et al. [24] in the case of the reassortants composed of PSV RNA1 and CMV RNA2 together with RNA3, and also by Dinant et al. [25] in the case of the combination of BMV 1a and CCMV 2a. More interestingly, we found that the reassortant T1C2C3 could infect plants efficiently, but was defective in production of RNA4A (Figure 1). The defect was restored partially when the C-terminal helicase domain in TAV 1a was replaced with that of CMV 1a (Figure 6), suggesting that the helicase domain played an important role in synthesis of RNA4A. The finding is in agreement with the previous report that capping activity of the methyltransferase domain of CMV 1a was not important for RNA transcription [42]. The core promoters of CMV RNA4 and RNA4A contain a similar stem-loop structure, which can compete for CMV replicase in vitro [43]. We found that the combination of TAV 1a and CMV 2a was ineffective in transcription of RNA4 and RNA4A, respectively, suggesting that TAV 1a may not effectively recognize the stem-loop structures of core promoters of CMV sgRNAs. Collectively, it might be common for the heterologous replicase complex to be deficient or defective in transcribing sgRNAs in the members of the family *Bromoviridae*.

## References

[1] Palukaitis P., García-Arenal F. (2003). Cucumoviruses. Adv. Virus Res..

[2] Ahlquist P., Dasgupta R., Kaesberg P. (1981). Near identity of 3- RNA secondary structure in bromoviruses and cucumber mosaic virus. Cell.

[3] Symons R.H. (1975). Cucumber mosaic virus RNA contains 7-methyl guanosine at the 5′-terminus of all four RNA species. Mol. Biol. Rep..

[4] Jacquemond M. (2012). Cucumber mosaic virus. Adv. Virus Res..

[5] Ahlquist P., Noueiry A.O., Lee W.M., Kushner D.B., Dye B.T. (2003). Host factors in positive-strand RNA virus genome replication. J. Virol..

[6] Kao C.C. (2002). Lessons learned from the core RNA promoters of Brome mosaic virus and Cucumber mosaic virus. Mol. Plant Pathol..

[7] Kim S.H., Palukaitis P., Park Y.I. (2002). Phosphorylation of cucumber mosaic virus RNA polymerase 2a protein inhibits formation of replicase complex. EMBO J..

[8] O’Reilly E.K., Tang N., Ahlquist P., Kao C.C. (1995). Biochemical and genetic analyses of the interaction between the helicase-like and polymerase-like proteins of the brome mosaic virus. Virology.

[9] Ding S.W., Anderson B.J., Haase H.R., Symons R.H. (1994). New overlapping gene encoded by the cucumber mosaic virus genome. Virology.

[10] Shimura H., Masuta C., Yoshida N., Sueda K., Suzuki M. (2013). The 2b protein of Asparagus virus 2 functions as an RNA silencing suppressor against systemic silencing to prove functional synteny with related cucumoviruses. Virology.

[11] Xin H.W., Ji L.H., Scott S.W., Symons R.H., Ding S.W. (1998). Ilarviruses encode a Cucumovirus-like 2b gene that is absent in other genera within the *Bromoviridae*. J. Virol..

[12] Diaz-Pendon J.A., Li F., Li W.X., Ding S.W. (2007). Suppression of antiviral silencing by cucumber mosaic virus 2b protein in Arabidopsis is associated with drastically reduced accumulation of three classes of viral small interfering RNAs. Plant Cell.

[13] Duan C.G., Fang Y.Y., Zhou B.J., Zhao J.H., Hou W.N., Zhu H., Ding S.W., Guo H.S. (2012). Suppression of Arabidopsis ARGONAUTE1-mediated slicing, transgene-induced RNA silencing, and DNA methylation by distinct domains of the Cucumber mosaic virus 2b protein. Plant Cell.

[14] Goto K., Kobori T., Kosaka Y., Natsuaki T., Masuta C. (2007). Characterization of silencing suppressor 2b of cucumber mosaic virus based on examination of its small RNA-binding abilities. Plant Cell Physiol..

[15] Du Z.Y., Chen F.F., Liao Q.S., Zhang H.R., Chen Y.F., Chen J.S. (2007). 2b ORFs encoded by subgroup IB strains of cucumber mosaic virus induce differential virulence on *Nicotiana* species. J. Gen. Virol..

[16] Gonzalez I., Martinez L., Rakitina D.V., Lewsey M.G., Atencio F.A., Llave C., Kalinina N.O., Carr J.P., Palukaitis P., Canto T. (2010). Cucumber mosaic virus 2b protein subcellular targets and interactions: Their significance to RNA silencing suppressor activity. Mol. Plant Microbe Interact..

[17] Gonzalez I., Rakitina D., Semashko M., Taliansky M., Praveen S., Palukaitis P., Carr J.P., Kalinina N., Canto T. (2012). RNA binding is more critical to the suppression of silencing function of Cucumber mosaic virus 2b protein than nuclear localization. RNA.

[18] Cillo F., Mascia T., Pasciuto M.M., Gallitelli D. (2009). Differential effects of mild and severe Cucumber mosaic virus strains in the perturbation of MicroRNA-regulated gene expression in tomato map to the 3′ sequence of RNA 2. Mol. Plant Microbe Interact..

[19] Du Z., Chen A., Chen W., Westwood J.H., Baulcombe D.C., Carr J.P. (2014). Using a Viral Vector to Reveal the Role of MicroRNA159 in Disease Symptom Induction by a Severe Strain of Cucumber mosaic virus. Plant Physiol..

[20] Lewsey M., Robertson F.C., Canto T., Palukaitis P., Carr J.P. (2007). Selective targeting of miRNA-regulated plant development by a viral counter-silencing protein. Plant J..

[21] Zhang X., Yuan Y.R., Pei Y., Lin S.S., Tuschl T., Patel D.J., Chua N.H. (2006). Cucumber mosaic virus-encoded 2b suppressor inhibits Arabidopsis Argonaute1 cleavage activity to counter plant defense. Genes Dev..

[22] Allison R.F., Janda M., Ahlquist P. (1988). Infectious in vitro transcripts from cowpea chlorotic mottle virus cDNA clones and exchange of individual RNA components with brome mosaic virus. J. Virol..

[23] Rao A.L., Francki R.I. (1981). Comparative studies on tomato aspermy and cucumber mosaic viruses. VI. Partial compatibility of genome segments from the two viruses. Virology.

[24] Suzuki M., Yoshida M., Yoshinuma T., Hibi T. (2003). Interaction of replicase components between Cucumber mosaic virus and Peanut stunt virus. J. Gen. Virol..

[25] Dinant S., Janda M., Kroner P.A., Ahlquist P. (1993). Bromovirus RNA replication and transcription require compatibility between the polymerase- and helicase-like viral RNA synthesis proteins. J. Virol..

[26] Salanki K., Carrere I., Jacquemond M., Balazs E., Tepfer M. (1997). Biological properties of pseudorecombinant and recombinant strains created with cucumber mosaic virus and tomato aspermy virus. J. Virol..

[27] Masuta C., Ueda S., Suzuki M., Uyeda I. (1998). Evolution of a quadripartite hybrid virus by interspecific exchange and recombination between replicase components of two related tripartite RNA viruses. Proc. Natl. Acad. Sci. USA.

[28] Feng J., Wang K., Liu X., Chen S., Chen J. (2009). The quantification of tomato microRNAs response to viral infection by stem-loop real-time RT-PCR. Gene.

[29] Wei S., Jin S.J., Zhang H., Wang T.W., Chen J., Liao Q. (2011). Sequences and Infectious Clones of Tomato aspermy virus Strain Isolated from Beijing. J. Agric. Biotechnol..

[30] Liao Q., Tu Y., Carr J.P., Du Z. (2015). An improved cucumber mosaic virus-based vector for efficient decoying of plant microRNAs. Sci. Rep..

[31] Rizzo T.M., Palukaitis P. (1990). Construction of full-length cDNA clones of cucumber mosaic virus RNAs 1, 2 and 3: Generation of infectious RNA transcripts. Mol. Gen. Genet..

[32] Weigel D., Glazebrook J. (2006). Transformation of agrobacterium using the freeze-thaw method. CSH Protoc..

[33] Silhavy D., Molnar A., Lucioli A., Szittya G., Hornyik C., Tavazza M., Burgyan J. (2002). A viral protein suppresses RNA silencing and binds silencing-generated, 21- to 25-nucleotide double-stranded RNAs. EMBO J..

[34] McGarvey P., Tousignant M., Geletka L., Cellini F., Kaper J.M. (1995). The complete sequence of a cucumber mosaic virus from Ixora that is deficient in the replication of satellite RNAs. J. Gen. Virol..

[35] Du Z., Chen A., Chen W., Liao Q., Zhang H., Bao Y., Roossinck M.J., Carr J.P. (2014). Nuclear-cytoplasmic partitioning of the Cucumber mosaic virus 2b protein determines the balance between its roles as a virulence determinant and RNA silencing suppressor. J. Virol..

[36] Naylor M., Murphy A., Berry J., Carr J. (1998). Salicylic acid can induce resistance to plant virus movement. Mol. Plant Microbe Interact..

[37] Shi B.J., Ding S.W., Symons R.H. (1997). In vivo expression of an overlapping gene encoded by the cucumoviruses. J. Gen. Virol..

[38] Soards A.J., Murphy A.M., Palukaitis P., Carr J.P. (2002). Virulence and differential local and systemic spread of cucumber mosaic virus in tobacco are affected by the CMV 2b protein. Mol. Plant Microbe Interact..

[39] Choi Y., Kang M.-Y., Lee J.-H., Kang W.-H., Hwang J., Kwon J.-K., Kang B.-C. (2016). Isolation and Characterization of Pepper Genes Interacting with the CMV-P1 Helicase Domain. PLoS ONE.

[40] Kang W.-H., Seo J.-K., Chung B.N., Kim K.-H., Kang B.-C. (2012). Helicase Domain Encoded by Cucumber mosaic virus RNA1 Determines Systemic Infection of Cmr1 in Pepper. PLoS ONE.

[41] Nguyen L., Lucas W.J., Ding B., Zaitlin M. (1996). Viral RNA trafficking is inhibited in replicase-mediated resistant transgenic tobacco plants. Proc. Natl. Acad. Sci. USA.

[42] Seo J.K., Kwon S.J., Choi H.S., Kim K.H. (2009). Evidence for alternate states of Cucumber mosaic virus replicase assembly in positive- and negative-strand RNA synthesis. Virology.

[43] Sivakumaran K., Chen M.H., Roossinck M.J., Kao C.C. (2002). Core promoter for initiation of Cucumber mosaic virus subgenomic RNA4A. Mol. Plant Pathol..

